# Books and Authors

**Published:** 1911-11

**Authors:** 


### BOOKS AND AUTHORS

**A Pocket Medical Dictionary, George M. Gould,** A.M., M.D., Ithaca, N. Y.; sixth edition, 34,000 words, P. Blakiston's Son & Co., Philadelphia. $1.00.

This handy little limp-leather pocket companion has been so favorably known in its earlier editions, that it is scarcely necessary to do more than mention the fact that a new edition has been required, by the exhaustion of older ones. Words are the pegs on which thoughts are hung. The student or physician who lets an unknown word pass him, throws a cloud over his understanding of an entire subject. Looking back over our first year of medical study, we recall that it was largely devoted to learning the meaning of new words. At the time, it seemed that nothing was really learned of medicine but, later, it was appreciated that merely the dictionary—and in those days, a convenient companion like the present, was practically unknown—had laid a good foundation for later courses.

---

**Cesare Lombroso, A Modern Man of Science, Hans Kurella,** M.D., translated from the German by **M. Eden Paul,** M.D.; published by the Rebman Co., New York, 1911. 194 pages including index, $1.50 cloth.

This is not merely a biography but rather a review of Lombroso's scientific work in criminal anthropology and psychology, pellagra, etc., and of the discussions of these important subjects by various critics. It should be read, not only by the neurologist, but by the criminal lawyer, the sociologist and philanthropist and by every man, physician or not, who wants to keep abreast of modern thought. The Rebman Company take special pride in their paper. There is no eye-strain from glistening surfaces, the book opens with flat pages requiring no readjustment of focus in following the line, and, on first taking up the book after being accustomed to the heavily impregnated paper used by so many publishers, the hand flies up. The labor of reading is thus lightened and there is not the temptation to let the book lie in the lap and bend over it to the detriment of the eyes.

---

**The Practical Medicine Series** (10 volumes annually). Vol. 6, General Medicine: General Editors for the Series, **Drs. Gustavus P. Head** and **Charles L. Minx;** Editors for this volume (as well as for Vol. 1 on the same subject) **Drs. Frank Billings** and **J. H. Salisbury.** The Year Book Publishers, Chicago, 1911. 353 pages, illustrated. Price for this volume, $1.50, for the series of 10 volumes, $10.

We have already reviewed previous volumes of this series which does so well for the busy practitioner and student, the work of sifting the wheat from the chaff of current medical literature. We note with pleasure the quotations of two physicians in our territory: Dr. Henry L. Elsner's (Syracuse), treatment of catarrhal jaundice: and E. Fuld's rediscovery, or rather independent discovery of A. L. Benedict's effervescence test for gastric acidity, with the manly recognition of the priority by Fuld.

**Diseases of the Stomach, with Special Reference to Treatment, Charles D. Aaron,** M.D., Detroit; Lea & Febiger, Philadelphia and New York, 1911. 555 pages, 21 plates and 42 other illustrations.

It is with a feeling of especial personal interest that we read this valuable addition to medical literature, as Dr. Aaron was a Buffalo boy, a graduate of the University of Buffalo and as we were his preceptor. Dr. Aaron very wisely follows the customary arrangement of previous books on the stomach, instead of attempting to give an impression of novelty by putting things out of their natural order. The true originality of the book appears all the more forcibly to the systematic reader. Dr. Aaron's belt for nephroptosis and enteroptosis, his work on epigastric herniæ and numerous minor points strike the eye as the reading progresses. The scope of the work is somewhat broader than the title, faecal analyses and duodenal investigation, being included. The colored plates illustrating gastric analysis are very convenient though we may say that the end reaction with alizarin does not give quite the tint that we see in an opaque, white porcelain titration dish, which we prefer to transparent beakers for all such work. Dr. Aaron, in common with most authorities, reads for free HCI at the complete color change with di-methyl-amido-azo-benzol, whereas we hold with the support of the minority, that the true end point for free acid is at the change from red to orange, about 10 degrees below the end-reaction.

These minor points of difference detract in no way from our high appreciation of the book and we always reserve the right to a change of view, as evidence accumulates. We commend this book to the use of the profession. Dr. Aaron, Dr. Buchmann and the editor originated the call which led to the organization of the American Gastro-enterologic Association in 1897 and Dr. Aaron has been secretary of that body continuously until the present year when his reiterated request for relief was finally granted with regret. Dr. Aaron's reputation is more than national and Buffalo, as well as Detroit, may well be proud of him.

---

**A Manual of Practical Hygiene,** for Students, Physicians and Health Officers, by the late **Dr. Charles Harrington,** 4th edition by **Dr. Mark Wyman Richardson;** Lea & Febiger, Philadelphia and New York, 1911. 850 pages, 12 plates and 124 engravings. $4.50.

One is fairly overwhelmed in attempting to review this collocal work and it may not be out of place to call attention to its cheapness merely from the standpoint of quantity, without regard to its high standard of quality. The first 268 pages comprise a very good substitute for the average work on dietetics, though the particular attention paid to analyses, detection of adulterants, etc., leaves a place for books dealing especially with dietetic therapeutics. Air, the soil, water, sewerage, construction of buildings, disinfection, etc., bring us to page 616. We then find a very complete treatise on military and naval hygiene, tropical hygiene, the relation of insects to disease, occupation in its relation to health, a brief but scholarly criticism of vital statistics, pointing out many fallacies which the ordinary student does not realize, a section on personal hygiene, a lucid discussion of immunity and various practical considerations of vaccination, quarantine, etc., and the book properly ends with a treatise on the disposal of the dead. In this last section, we note the increasing popularity of cremation and the fact that the Buffalo Crematory was among the earliest in the country.

The purchaser of this work is getting, not a book but a library.

---

**A Manual of Clinical Diagnosis** by **Charles E. Simon,** M.D., Baltimore; Lea & Febiger, Philadelphia and New York, 1911. Seventh edition, 778 pages, 25 plates, 168 engravings, $5.00.

The need for this new and enlarged edition is based both on the steady growth of medical science, largely due to the author, and on the equally steady demand for Simon's work, requiring either a new edition or a reissue of the previous edition. In the majority of cases, the popularity of a book depends upon the reputation and prestige of its author, even sometimes upon a position which implies a market. It would be putting the cart before the horse to say that Simon's first edition made him, but there have been few instances in the history of medical publication, in which a book sold so widely and so rapidly on its intrinsic merits, in which a book developed instead of following upon prestige.

While, for the sake of compactness, the author has left a field for treatises dealing specifically with the urine, blood, stomach contents, etc., his work is no student's manual or trite assemblage of clinical tests. Thoroughness and progress may be read between the lines. We like, too, the repeated dedication to his wife, in acknowledgement of her help.

